# Supplementary material for: Role and mechanism of NCAPD3 in promoting malignant behaviors in gastric cancer
Source: Front Pharmacol. 2024 Apr 22;15:1341039. doi: 10.3389/fphar.2024.1341039 (PMC11070777; doi:10.3389/fphar.2024.1341039)
Supplement: Supplementary file 11 [file DataSheet2.ZIP › GSEA/Transcipt factor/my_analysis.Gsea.1599461814734/DLX4_TARGET_GENES.html]

Details for gene set DLX4\_TARGET\_GENES[GSEA]

|  || Dataset | filtered\_dataset.sample\_info.cls#WT\_versus\_NCAPD3\_MUT |
| Phenotype | sample\_info.cls#WT\_versus\_NCAPD3\_MUT |
| Upregulated in class | WT |
| GeneSet | DLX4\_TARGET\_GENES |
| Enrichment Score (ES) | 0.26902115 |
| Normalized Enrichment Score (NES) | 1.8080716 |
| Nominal p-value | 0.0015527951 |
| FDR q-value | 0.31409553 |
| FWER p-Value | 0.976 |
Table: GSEA Results Summary

  

Fig 1: Enrichment plot: DLX4\_TARGET\_GENES      
 Profile of the Running ES Score & Positions of GeneSet Members on the Rank Ordered List

  

| SYMBOL | TITLE | RANK IN GENE LIST | RANK METRIC SCORE | RUNNING ES | CORE ENRICHMENT || 1 | 2120 | ETV6 | 63 | 0.862 | -0.0299 | Yes |
| 2 | 27304 | MOCS3 | 76 | 0.844 | -0.0215 | Yes |
| 3 | 153769 | SH3RF2 | 82 | 0.824 | -0.0082 | Yes |
| 4 | 112840 | WDR89 | 96 | 0.798 | -0.0015 | Yes |
| 5 | 50861 | STMN3 | 126 | 0.777 | -0.0073 | Yes |
| 6 | 51512 | GTSE1 | 127 | 0.774 | 0.0087 | Yes |
| 7 | 163882 | CNST | 140 | 0.757 | 0.0153 | Yes |
| 8 | 339318 | ZNF181 | 146 | 0.740 | 0.0269 | Yes |
| 9 | 51174 | TUBD1 | 153 | 0.730 | 0.0374 | Yes |
| 10 | 11162 | NUDT6 | 162 | 0.716 | 0.0462 | Yes |
| 11 | 817 | CAMK2D | 165 | 0.710 | 0.0594 | Yes |
| 12 | 51018 | RRP15 | 192 | 0.685 | 0.0539 | Yes |
| 13 | 348801 | LNP1 | 214 | 0.664 | 0.0518 | Yes |
| 14 | 79664 | ICE2 | 217 | 0.662 | 0.0640 | Yes |
| 15 | 54801 | HAUS6 | 219 | 0.660 | 0.0769 | Yes |
| 16 | 57521 | RPTOR | 239 | 0.645 | 0.0759 | Yes |
| 17 | 6426 | SRSF1 | 243 | 0.637 | 0.0868 | Yes |
| 18 | 5701 | PSMC2 | 244 | 0.636 | 0.1000 | Yes |
| 19 | 1161 | ERCC8 | 255 | 0.625 | 0.1054 | Yes |
| 20 | 87178 | PNPT1 | 263 | 0.622 | 0.1130 | Yes |
| 21 | 25974 | MMACHC | 265 | 0.621 | 0.1251 | Yes |
| 22 | 116143 | WDR92 | 267 | 0.619 | 0.1372 | Yes |
| 23 | 23341 | DNAJC16 | 276 | 0.613 | 0.1438 | Yes |
| 24 | 80124 | VCPIP1 | 282 | 0.607 | 0.1526 | Yes |
| 25 | 25852 | ARMC8 | 307 | 0.588 | 0.1466 | Yes |
| 26 | 7565 | ZNF17 | 316 | 0.585 | 0.1527 | Yes |
| 27 | 2342 | FNTB | 344 | 0.570 | 0.1440 | Yes |
| 28 | 9617 | MTRF1 | 349 | 0.568 | 0.1528 | Yes |
| 29 | 2965 | GTF2H1 | 356 | 0.563 | 0.1599 | Yes |
| 30 | 51535 | PPHLN1 | 361 | 0.560 | 0.1685 | Yes |
| 31 | 1962 | EHHADH | 392 | 0.541 | 0.1570 | Yes |
| 32 | 54554 | WDR5B | 395 | 0.539 | 0.1666 | Yes |
| 33 | 5814 | PURB | 399 | 0.536 | 0.1754 | Yes |
| 34 | 11019 | LIAS | 409 | 0.533 | 0.1797 | Yes |
| 35 | 147007 | TMEM199 | 410 | 0.533 | 0.1907 | Yes |
| 36 | 9743 | ARHGAP32 | 413 | 0.528 | 0.2002 | Yes |
| 37 | 54931 | TRMT10C | 446 | 0.507 | 0.1864 | Yes |
| 38 | 200933 | FBXO45 | 456 | 0.500 | 0.1900 | Yes |
| 39 | 23383 | MAU2 | 459 | 0.500 | 0.1988 | Yes |
| 40 | 5245 | PHB | 481 | 0.486 | 0.1930 | Yes |
| 41 | 53944 | CSNK1G1 | 485 | 0.484 | 0.2007 | Yes |
| 42 | 140775 | SMCR8 | 487 | 0.480 | 0.2099 | Yes |
| 43 | 130502 | TTC32 | 517 | 0.465 | 0.1976 | Yes |
| 44 | 1200 | TPP1 | 536 | 0.454 | 0.1934 | Yes |
| 45 | 84081 | NSRP1 | 538 | 0.453 | 0.2020 | Yes |
| 46 | 55527 | FEM1A | 543 | 0.449 | 0.2083 | Yes |
| 47 | 9836 | LCMT2 | 549 | 0.448 | 0.2138 | Yes |
| 48 | 9416 | DDX23 | 553 | 0.445 | 0.2207 | Yes |
| 49 | 5198 | PFAS | 570 | 0.437 | 0.2177 | Yes |
| 50 | 57579 | FAM135A | 577 | 0.436 | 0.2222 | Yes |
| 51 | 55245 | UQCC1 | 580 | 0.434 | 0.2297 | Yes |
| 52 | 26057 | ANKRD17 | 589 | 0.431 | 0.2325 | Yes |
| 53 | 9653 | HS2ST1 | 591 | 0.429 | 0.2407 | Yes |
| 54 | 55039 | TRMT12 | 599 | 0.424 | 0.2442 | Yes |
| 55 | 55181 | SMG8 | 602 | 0.422 | 0.2514 | Yes |
| 56 | 56339 | METTL3 | 613 | 0.417 | 0.2524 | Yes |
| 57 | 55308 | DDX19A | 615 | 0.416 | 0.2603 | Yes |
| 58 | 56252 | YLPM1 | 665 | 0.394 | 0.2314 | Yes |
| 59 | 26747 | NUFIP1 | 666 | 0.394 | 0.2395 | Yes |
| 60 | 57493 | HEG1 | 683 | 0.386 | 0.2354 | Yes |
| 61 | 55157 | DARS2 | 686 | 0.385 | 0.2419 | Yes |
| 62 | 9037 | SEMA5A | 688 | 0.385 | 0.2491 | Yes |
| 63 | 285636 | C5orf51 | 692 | 0.382 | 0.2548 | Yes |
| 64 | 89910 | UBE3B | 693 | 0.381 | 0.2627 | Yes |
| 65 | 10452 | TOMM40 | 696 | 0.380 | 0.2690 | Yes |
| 66 | 83743 | GRWD1 | 720 | 0.364 | 0.2591 | No |
| 67 | 134430 | WDR36 | 733 | 0.359 | 0.2575 | No |
| 68 | 64087 | MCCC2 | 747 | 0.352 | 0.2549 | No |
| 69 | 58509 | CACTIN | 748 | 0.351 | 0.2622 | No |
| 70 | 55178 | RNMTL1 | 771 | 0.337 | 0.2525 | No |
| 71 | 54939 | COMMD4 | 777 | 0.335 | 0.2557 | No |
| 72 | 9238 | TBRG4 | 786 | 0.330 | 0.2565 | No |
| 73 | 9343 | EFTUD2 | 818 | 0.305 | 0.2393 | No |
| 74 | 54968 | TMEM70 | 879 | -0.291 | 0.1999 | No |
| 75 | 23412 | COMMD3 | 894 | -0.312 | 0.1958 | No |
| 76 | 54532 | USP53 | 898 | -0.322 | 0.2001 | No |
| 77 | 440026 | TMEM41B | 932 | -0.355 | 0.1825 | No |
| 78 | 3475 | IFRD1 | 935 | -0.360 | 0.1885 | No |
| 79 | 153830 | RNF145 | 940 | -0.363 | 0.1930 | No |
| 80 | 55186 | SLC25A36 | 969 | -0.387 | 0.1798 | No |
| 81 | 3176 | HNMT | 991 | -0.398 | 0.1721 | No |
| 82 | 5889 | RAD51C | 1059 | -0.451 | 0.1307 | No |
| 83 | 9939 | RBM8A | 1093 | -0.474 | 0.1155 | No |
| 84 | 9245 | GCNT3 | 1147 | -0.511 | 0.0860 | No |
| 85 | 55124 | PIWIL2 | 1154 | -0.516 | 0.0921 | No |
| 86 | 8345 | HIST1H2BH | 1207 | -0.585 | 0.0648 | No |
| 87 | 10802 | SEC24A | 1251 | -0.639 | 0.0455 | No |
| 88 | 135114 | HINT3 | 1257 | -0.645 | 0.0551 | No |
| 89 | 8412 | BCAR3 | 1261 | -0.648 | 0.0663 | No |
| 90 | 26959 | HBP1 | 1395 | -1.073 | -0.0123 | No |
| 91 | 8548 | BLZF1 | 1398 | -1.103 | 0.0091 | No |
Table: GSEA details [plain text format]

  

Fig 2: DLX4\_TARGET\_GENES      
 Blue-Pink O' Gram in the Space of the Analyzed GeneSet

  

Fig 3: DLX4\_TARGET\_GENES: Random ES distribution      
 Gene set null distribution of ES for **DLX4\_TARGET\_GENES**
